# Innovations in Cattle Breeding Technology: Prospects in the Era of Gene Editing

**DOI:** 10.3390/ani15101364

**Published:** 2025-05-08

**Authors:** Yu Wang, Xiangshun Cui, Zhi Chen

**Affiliations:** 1 College of Animal Science and Technology, Yangzhou University, Yangzhou 225009, China; 221902118@stu.yzu.edu.cn; 2 Laboratory of Animal Developmental Biology, Department of Animal Science, Chungbuk National University, Cheongju 28644, Republic of Korea; xscui@cbnu.ac.kr

**Keywords:** cattle, breeding, gene editing, biotechnology

## Abstract

As a core species in the global livestock industry, cattle play an irreplaceable role in human food security and economic development. Beef cattle and dairy cattle meet the dietary needs of billions of people around the world by providing high-quality protein and dairy products, respectively. With the growth in population and the intensification of the pressure of climate change, traditional breeding techniques may be unsuitable to meet the increasingly growing demands for sustainable and highly adaptable processes. In recent years, the rapid development of genomics, bioinformatics, and gene-editing technologies has provided unprecedented tools and perspectives for the genetic improvement of cattle, driving the precise design and efficient development of new cattle breeds. However, the development of new cattle breeds still faces multiple bottlenecks pertaining to scientific, ethical, and industrialization aspects, which can be addressed through interdisciplinary collaboration. In this review, we will systematically assess the technological progress in the genetic breeding of beef cattle and dairy cattle, analyze the integration path of traditional breeding and modern biotechnology, and explore the future directions of cattle breeding research under the sustainable development goals, with the aim of providing theoretical support for cattle breeding.

## Simple Summary

With the rapid advancement of modern society and the continuous growth of the global population, consumers’ demand for cattle and their products, including high-quality beef and dairy products, has been steadily increasing. However, the existing cattle breeds currently known to people are confronted with a multitude of production-related challenges. These issues range from low meat and milk production efficiency and susceptibility to diseases to suboptimal adaptation to diverse environmental conditions, all of which necessitate continuous efforts to enhance their traits in real-world production scenarios. To address these challenges, scholars are actively exploring new ways to breed novel cattle breeds. By introducing cutting-edge gene editing technology and in-depth analysis of candidate genes that impact various important traits of cattle, such as growth rate, disease resistance, and milk quality, we strive to provide practical and innovative ideas and methodologies for the successful cultivation of new cattle breeds.

## 1. Introduction

**Figure 1:**
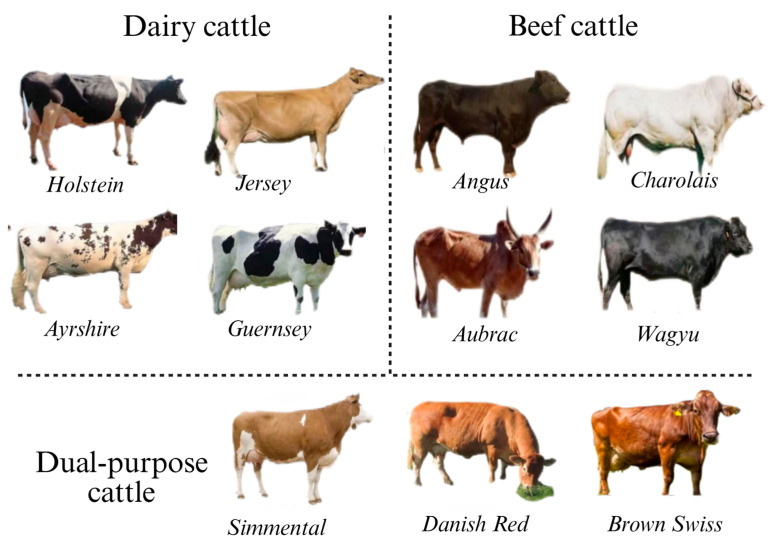
Common breeds of cattle in the world (including dairy cattle, beef cattle, and dual-purpose cattle).

The demand for beef and dairy products continues to increase, driven by the global population growth and rising living standards [1]. Cattle, as one of the most important domestic animals, hold an irreplaceable position in human society. Globally, several breeds of dairy and beef cattle are indispensable in the livestock industry due to their distinct genetic traits, superior production performance, and substantial economic value. Dairy breeds are renowned for their high milk production or superior milk quality. Among them, the *Holstein breed* stands out as the most globally widespread breed, easily recognizable by its distinctive black-and-white patches and renowned for its high average annual milk yield. In contrast, the *Jersey breed*, though smaller in size, is prized for its exceptionally high milk fat content [2]. The *Ayrshire breed* thrives in hilly pasture environments, and the *Guernsey breed* is renowned for the high carotenoid content of its milk, which contributes to its distinctive richness. Beef breeds are distinguished by their core strengths in terms of growth rate, muscle development, and meat quality. The *Angus breed* is globally celebrated for its hornless black coat, exceptional marbling, and tender meat. The *Charolais cattle* from France are known for their large size and rapid weight gain, making them ideal for intensive fattening programs. The *Aubrac cattle*, prized for their juiciness and tenderness, are another notable breed. Meanwhile, the *Japanese Wagyu cattle* stand at the pinnacle of beef production, renowned for their extreme marbling and unique, rich flavor profile. In addition, multipurpose breeds, such as the *Swiss Simmental* and *Danish Red*, integrate both milk and meat production capabilities, offering versatile options for diversified farming systems. Through continuous selective breeding and crossbreeding efforts, these breeds have significantly contributed to the advancement and efficiency of modern animal husbandry practices (Figure 1).

The development of new cattle breeds is crucial for driving the progress of the livestock industry, meeting evolving market demands, and enhancing the industry’s overall competitiveness [3]. High-quality cattle breeds are the key to improving farming efficiency and production levels; however, dairy cattle, beef cattle, and dual-purpose cattle breeds face their own diverse set of problems. Traditional cattle breeds often face limitations in terms of their growth rate, meat and milk production performance, and disease resistance, which hinder their ability to meet the demands of modern animal husbandry for scale, intensification, and high efficiency. As a critical component of modern animal husbandry, dairy cattle breeding encounters numerous challenges while striving to enhance milk production efficiency and economic returns. The declining genetic diversity is a core problem, and overreliance on a few high-yielding sires leads to increased inbreeding rates. The accumulation of recessive harmful genes may trigger risks such as reduced fertility in offspring [4], weakened immunity, and a prevalence of metabolic diseases. Secondly, achieving a balance between high production traits and health traits remains a significant challenge. An overemphasis on milk production may compromise a cow’s disease resistance, limb health, or longevity, ultimately undermining the long-term sustainability of the herd [5]. Ethical and biosafety controversies in the application of reproduction technologies such as embryo transfer and gene editing techniques, which may lead to the spread of genetic defects, compromise animal welfare, or produce unforeseen ecosystem chain reactions [6]. In addition, the tension between environmental adaptability and intensive farming practices is becoming increasingly pronounced. Many high-yielding dairy breeds exhibit significant sensitivity to specific climatic or feeding conditions, which limits their ability to adapt to climate change or meet the production demands of resource-constrained regions. The technological and germplasm barriers between different countries are one of the important factors contributing to the slow development of the beef cattle industry. For example, China has long relied on imported breeds (such as Simmental and Angus). However, due to insufficient local adaptive breeding efforts post-introduction, these breeds often experience performance degradation, trapping the industry in a cycle of ‘introduction–degradation–re-introduction’. Meanwhile, indigenous specialty breeds, such as Qinchuan cattle and Luxi cattle, are increasingly marginalized due to the lack of systematic selection and breeding programs. Additionally, the whole-genome databases for breeding cattle have insufficient coverage, and the reliance on imported performance measurement equipment further limits the accuracy of genetic evaluations. On the breeding front, small-scale free-range operations dominate, often lacking performance measurement systems and genealogical records. This results in a fragmented breeding data chain, compounded by an inadequate female stock, which collectively hinders the sustainability of continuous breeding efforts [7].

Gene editing is significant in the field of breeding new cattle lines [8]. Gene editing technology enables precise gene localization and modification, overcoming the limitations of traditional breeding methods, such as a long generation interval. This breakthrough significantly reduces the breeding time and accelerates the development of superior varieties. Additionally, gene editing unlocks and utilizes previously inaccessible genetic resources, facilitating targeted innovations that lead to higher yields and improved quality in new varieties. In addition, cattle breeding has fully entered the era of genomic selection [9]. GS can be applied to cattle breeding for predicting the precocious puberty of cows and selecting some traits of young bulls that are difficult to measure [10]. In addition, GS can also be applied in fields such as the paternity testing of cattle herds and the prediction of embryo genomes [11]. Furthermore, high-density genotyping is the basis for the genome-wide linkage analyses and genetic evaluation of cattle. The use of gene chips allows for easier, faster, and more accurate large-scale typing. A large number of studies have shown that the inclusion of functional loci that are significantly associated with target traits in gene chips can significantly improve the accuracy of genomic selection [12,13]. Gene chips act as a pivotal bridge connecting various technologies in biological breeding. In the realm of gene editing, they provide essential technological support. For screening and identifying gene targets, gene chips enable high-throughput genome-wide scanning, pinpointing the precise entry points for gene editing. When assessing gene editing efficiency, gene microarrays can detect changes in gene expression before and after editing, offering a comprehensive view of the editing outcomes. They also verify editing specificity and identify potential off-target risks. Furthermore, in studying gene editing mechanisms, gene microarrays facilitate a deeper understanding of intracellular signaling pathway remodeling and gene regulatory network dynamics by monitoring the behavior of relevant genes throughout the gene editing process.

New cattle lines can be developed by introducing or enhancing superior traits, such as improving the growth rates and meat quality of beef cattle, increasing the milk yield and quality of dairy cattle, and boosting the disease and environmental resilience of herds. These advancements contribute to the overall productivity and economic efficiency of the livestock industry, fostering its sustainable development. Therefore, this review will provide a brief overview of gene editing techniques commonly used in breeding and assess the potential candidate genes for developing new dairy and beef cattle lines. The aim of this review is to offer a theoretical foundation and practical insights for breeding innovative cattle varieties.

## 2. From CRISPR/Cas9 to Multi-Gene Editing: New Pathways for Genetic Improvement of Cattle

Gene editing is a genetic engineering activity that involves altering an organism’s specific genomic DNA by means of DNA sequence knockouts, insertions, and base editing to change its properties and functions [14]. Major gene editing technologies include zinc finger nucleases (ZFNs), transcription activator-like effector nucleases (TALENs), and clusters of regularly interspaced short palindromic repeats (CRISPR)/Cas9 gene editing [15,16].

Since the beginning of the 21st century, the most popular gene editing technology has been the CRISPR/Cas9 system. Some scholars have shown that the CRISPR/Cas9 system can be successfully applied in breeding to improve reproductive traits and production performance and introduce strong disease resistance traits in a variety of livestock. This can be achieved due to the CRISPR/Cas9 system’s advantages of a low application cost, a wide editing scope, a high targeting efficiency, simple operation, and support of multi-site operations [17]. Cattle are monozygotic animals, their reproduction cycle and growth cycle are longer than those of poultry, and the associated cost of experimentation is high. The development of gene editing is relatively lagging behind, and through the learning and application of CRISPR/Cas9 technology, we will be able to promote the rapid development of research in this field. However, despite the rapid development of gene editing technology, this technology is still limited to editing a single gene and improving a single trait, which no longer meets the current stage of breeding needs. Moreover, during the breeding process, some traits may be controlled by multiple genes [18]. In such circumstances, it is rather challenging to edit a single gene to change these traits. In order to resolve this situation, a simple and efficient multi-gene editing system is needed. The use of gene editing technology for the simultaneous editing or transcriptional regulation of multiple loci has become a major trend, which is of great significance for the improvement in livestock germplasm.

**Figure 2:**
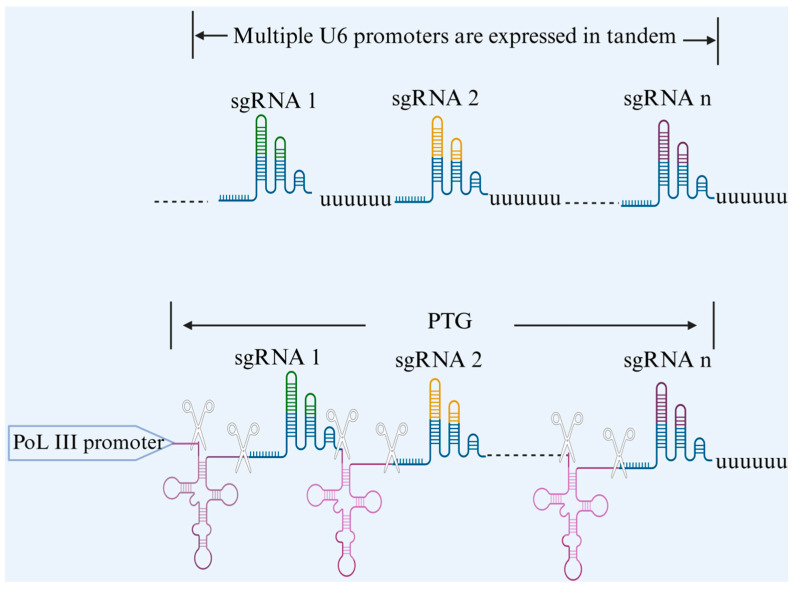
Schematic representation of multiple single cis–trans sgRNA expression cassettes versus multiple cis–trans sgRNA expression cassettes.

In multiple gene editing technology, we need to introduce two or more sgRNAs at the same time so that the Cas9 protein can be guided by multiple sgRNAs to effectively recognize multiple target sites [19]. Different scholars have given different answers to the question of how to introduce multiple sgRNAs at the same time [20]. The more common methods include the establishment of sgRNA expression boxes containing multiple monocistronic sgRNA expression boxes and the establishment of multicistronic sgRNA expression boxes. For multiple individual sgRNA expression cassettes, it is generally possible to tandemly connect multiple repetitive Pol III-type promoter-initiated sgRNA expression cassettes, each of which is transcribed by a separate RNA polymerase III (Pol III) promoter [21]. The common promoters in this system are U3 and U6, and these Pol III promoters start transcription at specific nucleotides. For example, the U3 promoter transcribes from ‘A’, and the U6 promoter transcribes from ‘G’ [22]. Although this method allows for the editing of multiple genes, the design of the system necessitates the separate assembly of promoters, terminators, and sgRNA backbones, which is not only cumbersome to execute and requires repetitive work but also affects each of the transcriptional activities of neighboring promoters [23]. Thus, multi-cis sgRNA expression cassettes are preferred today. For such systems, there are three common constructs: nuclease Csy4-mediated multi-cis sgRNA processing, tRNA-based multi-cis sgRNA processing, and self-cleaving nuclease-mediated multi-cis sgRNA processing. Next, we will briefly describe three ways of building multi-cis sgRNA expression cassettes. Csy4, a crRNA-processing protein from Pseudomonas aeruginosa, is a specific ribonucleic acid endonuclease. By artificially installing Csy4 excision sites on both sides of sgRNAs to construct a system of Cys4-processed-sgRNAs with a tandem structure in which Csy4 can cleave each of the repetitive sequences, the Csy4-mediated arrays of gRNAs can efficiently release a large number of sgRNAs at the same time [24,25,26]. As for the multi-cis-transon sgRNA processing of tRNAs, the 5′ and 3′ end sequences of tRNAs, which are present in every organism, can be specifically recognized and cleaved by the cellular endogenous RNaseP and RNaseZ enzymes, releasing independent tRNAs and mature sgRNAs that direct the Cas9 protein to cleave the target sites. By constructing an artificial multiple cis-tRNA-gRNA (PTG) gene tandem expression system with the tRNA-sgRNA-sgRNA backbone as the minimal repeating unit, it is possible to enable the efficient expression of multiple sgRNAs from a single Pol III promoter [27]. Since multiple gene editing using self-cleaving nucleases has been developed on a large scale in microorganisms and because there are very few reports of multiple sgRNA expression in animals using self-cleaving systems, we will not cover them in this review. Different multi-gene editing systems have their own specific constraints, yet they all face the same pressing problem: too many sgRNA species compete for the limited number of nucleases in a cell, a phenomenon that makes knockdown less efficient and causes more sgRNAs to be in tandem. However, we cannot deny that the simultaneous editing of multiple genes is a key breakthrough in the use of CRISPR gene editing technology to genetically breed new lines of cattle, which is of great significance to the field of livestock production (Figure 2).

Single-gene and multi-gene editing technologies collectively provide precise and efficient pathways for advancing cattle breeding. Single-gene editing enables the rapid introduction of specific traits by targeting key functional genes, while multi-gene editing addresses the genetic antagonism between traits through the synergistic regulation of complex genetic networks. This approach overcomes the bottleneck of optimizing multiple traits in traditional breeding methods. Some cattle breeds have already been modified through single-gene editing to confer heat-tolerance mutations, thereby enhancing their resistance to heat stress [28]. Additionally, there are cattle that have had their reproductive capabilities improved through single-gene editing [29]. Although there are not many reports showing the application of multi-gene editing in cattle breeding as of today, i.e., up to 2025, it has already achieved an adequate level of success in the poultry industry [30]. This bolsters our confidence in the application of this technology in cattle breeding. The combination of the two strategies not only accelerates the breeding of ‘customized’ cattle but also promotes the upgradation of the livestock sector towards efficiency and sustainability.

## 3. Breeding New Lines of Dairy Cattle

### 3.1. Selection of Key Genes in Dairy Cattle Breeding

If people want to breed new high-quality cows, they must pay attention to the expression of relevant genes that can affect the characteristics of the progenitor cows. The number of genes that can influence lactation and reproduction traits in dairy cows is very large (Table 1) and cannot be exhaustively reviewed in this review. Therefore, this review will focus on several gene families widely recognized by the academic community for their roles in lactation initiation, milk fat synthesis, and milk fat transport in dairy cows. This review will summarize their known functions and explore their potential applications.

#### 3.1.1. *DGAT*

Triglycerides are neutral lipids and are the main energy storage lipids in mammals and are essential substances for increasing the amount of milk fat in dairy cows. *DGAT1* and *DGAT2* have long been found to be ubiquitously expressed in mammalian tissues, with the highest levels of expression in tissues with active triglyceride synthesis, such as adipose tissue, the small intestine, liver, and mammary gland. Both have been shown to catalyze the final step of triglyceride synthesis and are involved in the formation of triglycerides [48]. Although both *DGAT1* and *DGAT2* have the acyl coenzyme A–glycerol diester acyltransferase activity, they have no sequence homology, belonging to different gene families and exhibiting different biochemical, cellular, and physiological functions [49]; thus, we need to treat both of them differently when exploring their role in cattle breeding.

We focus on the *DGAT1* gene, located on bovine chromosome 14, which is a key gene affecting milk production traits in a wide range of cattle breeds [50]. It has been shown that the exonic region of the *DGAT1* gene causes different dairy breeds to exhibit different milk production traits [51]. For example, the g.8525C>T SNP genotype of the *DGAT1* gene showed a highly significant association with milk proteins in Bangladeshi river buffaloes [52], whereas the same SNP genotype (g.8525C>T) showed a significant correlation with milk fat in the Riverside buffalo population [53]; thus, its role in Holstein cows needs to be further explored. In addition, mutations of only *DGAT1* play a very important role in milk production in dairy cows. The non-synonymous mutation K232A in exon 8 of *DGAT1* leads to a change in the encoded amino acid from lysine to alanine, which alters the yield and composition of milk [54] and is also associated with a higher fat and protein content [55]. In addition, the K232A mutation has been reported to have a positive effect on the clotting ability of the milk of *Holstein cows* from some locations [56], which has led us to focus on the utilization of this mutation when breeding new Holstein cows. Moreover, differences in the genotype of *DGAT1* may also alter the lipid composition of the milk fat globule membrane, changing its PL/TAG ratio and, thus, affecting the amount of milk fat [57].

Focusing on the role of *DGAT1* in cow lactation, researchers cannot ignore this key gene, as it is considered an immune modulator [58]. Another powerful function of *DGAT1* is to protect cells from the pro-inflammatory effects and lipotoxicity of lipids while promoting lipid accumulation with immunoactivity regulation [48]. Therefore, *DGAT1* can effectively reduce the probability of mastitis in new *Holstein cows*, which is highly favorable for the breeding and promotion of new breeds.

#### 3.1.2. *GHR*

Previous studies have shown that growth hormone (GH) plays a significant role in lactogenesis, and the growth hormone receptor (GHR), which can mediate its action, is considered a functional candidate gene for milk performance in dairy cows [59]. Through some of its functions, we know that *GHR* is abundantly expressed on the membrane and in the cytoplasm of bovine mammary epithelial cells (BMECs) [60]. The bovine *GHR* gene was also found to be localized on chromosome 20 and consists of 10 exons (E), of which E1 is very small and has a non-coding sequence [61,62].

Interestingly, similar to *DGAT*, genetic polymorphisms in *GHR* also influence lactation yield, milk fat, milk protein, and other relevant contents of cow’s milk [63,64]. When a single-nucleotide substitution from T to A occurs in exon 8 of the *GHR* gene, it results in the substitution of a neutral phenylalanine (279F) at position 279 of the amino acid sequence with an uncharged but polar tyrosine (279Y), a change that is located in the transmembrane structural domain of the *GHR* gene and has an impact on the milk yield of some dairy cow populations [65]. Based on this variation in milk yield, it has been found that the *GHR F279Y* genotype has a significant effect on the milk yield, protein and fat contents, casein production and content, and lactose production and content of the *German Holstein cow* population [66], and this genotype also has a positive effect on the milk production traits of *Chinese Holstein cows* [67]. The *GHR* gene also regulates the uptake and utilization of nutrients by mammary cells, increasing the supply of raw materials for milk synthesis, thus increasing milk production. From these studies, it is evident that the *GHR F279Y* genotype plays a significant role in dairy cattle populations from diverse places; thus, it is necessary to pay attention to this gene when attempting to breed new types of dairy cattle.

In addition, *GHR* can activate various signaling pathways, including JAK2/STAT, MAPK, and PI3K [68]. By activating the downstream signaling pathways related to milk synthesis, it can more effectively promote the uptake of nutrients by mammary cells for milk synthesis, thus increasing milk production. For example, the JAK-STAT5 pathway is the main pathway by which growth affects the expression of genes encoding milk proteins (e.g., β-casein); thus, *GHR* not only acts alone but also undertakes important tasks in different pathways involved in milk production [69], making it a more versatile gene. These pathways promote bone growth, muscle formation, and overall body development in young cows, helping them to achieve a suitable adult body shape and laying the foundation for subsequent performance.

#### 3.1.3. *PRL*

It is well known that the prolactin (*PRL*) gene is on chromosome 23 in cattle, spans 9.4 kb, and contains five exons and four introns, and it is not contained in any chain-group quantitative trait loci (QTLs).

*PRL* is not only a key regulator of mammalian reproductive processes but also is involved in breast proliferation and differentiation during pregnancy [70] and is a key gene in breast development [71]. Specifically, it is similar to the *GHR* gene, and the important pathway induced is the Jak-STAT signaling pathway [72]. *PRL* can also stimulate the STAT5 pathway to influence cell proliferation and differentiation [73], thus playing a role in the mammary gland development and milk production in animals. In the *PRL* gene studies of cattle conducted by other scholars, a variety of polymorphisms have been found in the bovine *PRL* gene [74]. To date, 45 SNPs located within exons have been recorded in the Ensembl Variation database. By exploring these SNPs, researchers have demonstrated that a common polymorphism within exon 4 of the bovine *PRL* gene (rs211032652 SNP, c.396G>A) can have a definite effect on the milk yield, milk protein, and milk fat of some dairy cattle populations [75].

Mastitis is a serious, unavoidable problem of dairy production, with implications for both public health and milk production [76]. The study of the effect of *PRL* on bovine mastitis resistance is a very interesting topic. For example, it has been found that *PRL* promotes the inflammatory response of bovine mammary epithelial cells through NF-kB activity [77,78]. However, it is crucial to assess whether polymorphisms in the *PRL* gene are related to the immune functions of dairy cows. Fortunately, some researchers have found a high correlation between genetic polymorphisms associated with prolactin and mastitis resistance and current dairy breeds’ susceptibility to mastitis [79]. This bolsters the importance of the *PRL* gene in dairy cattle breeding.

### 3.2. Outlook on Dairy Breeding

For animals with long generation intervals, such as dairy cows, genome selection using gene chip typing can greatly shorten the generation interval and accelerate the process of genetic improvement. The numerous studies mentioned earlier have identified genetic markers associated with economically important traits such as milk production, reproduction, growth, and health [31,36,38], which can provide technical and theoretical support for breeding new lines of dairy cattle. For example, Chinese scholars have previously reported GWASs for milk production traits in *Chinese Holstein cows* and identified 105 significantly associated SNP loci using the Illumina Bovine SNP50 Microarray [80]. By using gene chips to mine and tag genetic information related to milk production traits, we can select dairy breeds with a higher milk yield and a better milk composition, which can directly improve the economic benefits of dairy farming. Moreover, association analyses have been carried out using multiple SNP microarrays with adult Holstein heifers to assess their conception and pregnancy rates and with young Holstein heifers to assess their conception rate, identifying a number of validated genes, including GN-RHR, and new regions in the cluster of pregnancy-associated glycoprotein genes [81], which provide a reference for unraveling the genetic variation and genomic regions of the fertility traits of dairy cows. Genetic markers related to reproduction can help us to select cows with higher fertility and shorter reproduction cycles and to improve the reproductive efficiency of new lines of cows. In addition, the genes affecting the body size and the limbs and hooves of current cow breeds (e.g., *ADIPOR2*, *INPP4A*, *DNMT3A*), genes causing mastitis in current cow breeds (e.g., *ABCC9*, *ACHE*), and genes causing ketosis in current cow breeds (e.g., *NRXN3*, *ACOXL*, *BCL2L11*) have been investigated to a certain extent [82,83,84,85], which facilitates the selection and editing of genes for future studies.

**Figure 3:**
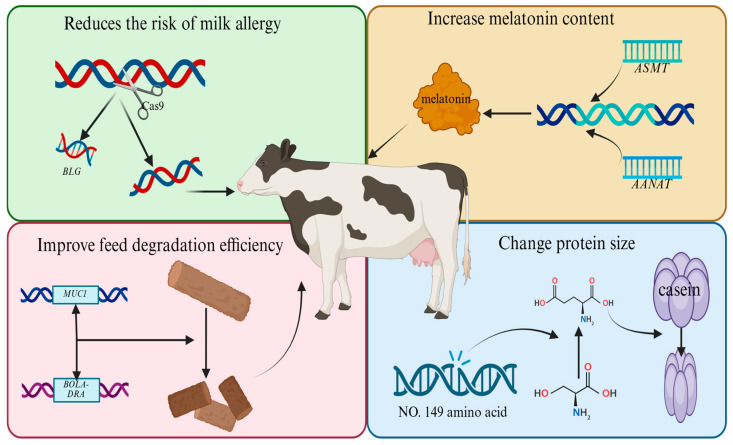
Schematic representation of breeding new lines of dairy cattle.

Scientists can use CRISPR/Cas9 to knock out the β-lactoglobulin gene (*BLG*) when breeding cows. β-lactoglobulin is an allergen found in milk, and knocking out *BLG* would make the milk free of this allergen. Editing *SCD1*, a key regulator of the fatty acid metabolic pathway, to produce low-fat milk or editing *ASMT* and *AANAT* to increase the amount of melatonin in milk are ideas to further improve the milk quality of new dairy cow breeds. For the breeding of new lines of dairy cattle in China, we can exploit the function of indigenous Sanhe cattle rumen microbial interactions (e.g., BOLA-DRA and *MUC1*) that significantly enhance the efficiency of roughage degradation and use the CRISPR-Cas12f system to achieve the simultaneous editing of multiple genes in order to breed feed-saving dairy cattle lines with a high efficiency of feed conversion. For the optimization of milk composition, we can first construct a model of the milk protein anabolic network and precisely regulate the κ-casein phosphorylation site (Ser149→Glu) using the base editing technology so as to reduce the particle sizes of casein micelles and significantly improve the processing characteristics of dairy products. In the field of disease resistance breeding, a real-time monitoring system for mastitis resistance based on SNP gene chips has been developed, which, in combination with genome in situ synthesis technology, integrates the lysostaphin gene into the mammary gland-specific expression locus in a targeted manner, reducing the incidence of clinical mastitis. The aforementioned methods can be used to establish programs for global dairy cattle breeding (Figure 3).

## 4. Breeding New Lines of Beef Cattle

### 4.1. Selection of Key Genes in Beef Cattle Breeding

Consumer perceptions of beef quality are shaped by both intrinsic attributes, such as nutritional value, and extrinsic attributes, including appearance and sensory characteristics. These factors play a pivotal role in influencing consumer preferences. Therefore, similar to dairy cattle breeding, it is essential to identify and understand the diverse range of genes that impact meat quality, tenderness, and other traits when developing new beef cattle lines (Table 2). Therefore, we summarize the candidate genes involved in the important traits of beef cattle breeding. This provides a basis for the development of new lines of beef cattle.

#### 4.1.1. *MSTN*

The genetic variation in the *MSTN* gene (also known as the *GDF8* gene), located on bovine chromosome 2, has been found to be associated with an increase in the number of muscle fibers in cattle, leading to more muscular animals or double-muscled animals [104]. When animals exhibit dual-muscle genotypes, they have low water-holding capacity (WHC), fat coverage, and fat content in the LMA (muscle cross-sectional area). Furthermore, double-muscled animals have high carcass yield and muscle weight and area. Although the reduced fat content and low WHC negatively affect the organoleptic attributes of meat compared to other genotypes with a higher fat content, because of their higher CW and body size, higher efficiency, and lower feeding costs (due to a better feed conversion), these animals provide higher economic returns in intensive production systems [105]. Moreover, a lower IMF and the different proportions of FAs are considered positive for human health. Compared to other animals with normal genotypes, herds with the dual-muscle genotype have lower total FAs, and they have a higher proportion of polyunsaturated fats (PUFAs) in their bodies, resulting in a higher polyunsaturated/saturated fat ratio and a lower n-6/n-3 ratio [106]. This double-muscled trait has been associated with reduced fertility [107]; thus, it is important to focus on the muscle growth rate of cattle in tandem with their reduced fertility.

#### 4.1.2. *CAPN* and *CAST*

*CAST* and *CAPN*, two of the most important genes for meat production and composition, are located in cattle on chromosomes 7 and 29, respectively, and they are associated with performances related to meat production traits. Meat tenderness is a prominent characteristic among the quality characteristics of meat that are most desired by consumers. Beef tenderness is associated with higher CSET concentrations. This is attributed to the inhibition of *CAPN* by *CAST* during post-mortem muscle protein hydrolysis [108], as the *CAST* gene leads to more calpain inhibitor proteins and, therefore, a higher rate of calpain inhibition. This suggests that *CAPN* is the main reason for the increase in meat tenderness during the post-slaughter process [109]. Another important reason for selecting *CAST* as a candidate gene is that *CAST* activity has a very high heritability and is closely related to meat shear, which means that meat tenderness can be improved in animals selected for *CAST*-based modifications; given the high heritability of *CAST*, a rapid genetic response can be achieved by selecting animals for CAST-based modifications [110].

#### 4.1.3. *LEP*

The *LEP* gene is located on chromosome 4 and encodes the gene for the leptin hormone, which acts on receptors in the hypothalamus controlled by the leptin receptor (*LEPR*) gene to suppress appetite. As such, it affects the regulation of feed intake, so it is related to body composition, energy balance, and body weight [91]. Furthermore, the serum leptin concentration is positively correlated with the marbling score, SF, and USDA quality grade, but it is negatively correlated with LMA [111]. These findings imply that leptin can be used as an indicator for assessing carcass composition in live animals and is useful in breeding programs.

### 4.2. Beef Cattle Breeding Expectations

**Figure 4:**
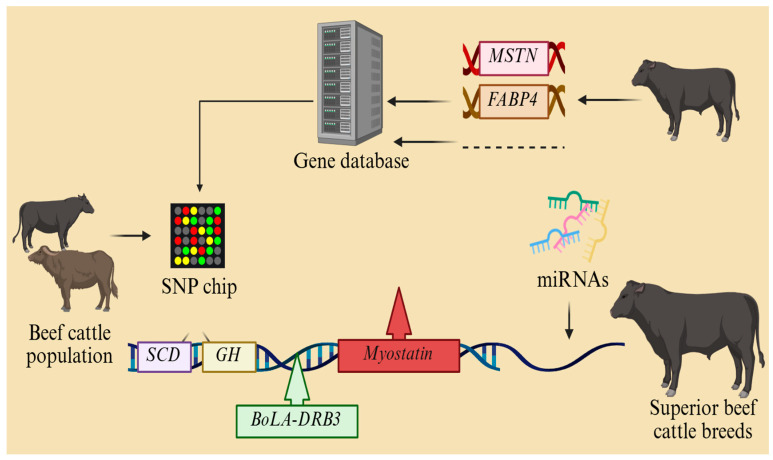
Schematic of breeding new lines of beef cattle.

Genetic technology innovation is the core driving force to overcome the bottleneck of traditional breeding in the breeding of new lines of beef cattle. Firstly, whole-genome sequencing technology should be used to systematically analyze the genetic characteristics of local cattle breeds (e.g., *Qinchuan* and *Nanyang cattle*) to explore the functional loci related to meat quality (e.g., the *FABP4* gene), growth rate (e.g., the *MSTN* gene), and resistance, and to set up a gene database. The early and precise selection of bulls through genomic selection (GS) technology shortens the selection cycle from the traditional 5–6 generations to 2–3 generations, dramatically advancing genetic progress. Secondly, CRISPR/Cas9 gene editing can be used to target key traits, such as knocking out the myostatin gene to promote muscle growth or introducing natural resistance genes (e.g., the *BoLA-DRB3* variant) to increase resistance to Mycoplasma bovis pneumonia. At the same time, SNP molecular marker-assisted breeding chips have been developed to implement polygenic selection for complex traits such as intramuscular fat deposition (e.g., the *SCD* gene) and feed conversion rate (e.g., the *GH* gene). In addition, it is necessary to strengthen the research on epigenetic regulatory mechanisms and to enhance the efficacy of gene expression by means of methylation modifications and miRNA interventions. Genomic selection technology can be used to screen breeders with the high feed conversion rate and high-quality marbling deposition to establish an efficient breeding system. Moreover, we can analyze gene–nutrient interactions through metabolomics and transcriptomics, develop customized feeding programs, and use single-cell sequencing to reveal the synergistic regulatory network between rumen microbes and host genes so as to select ecologically adapted lines for extreme environments, such as plateaus and hot and humid environments. Moreover, we should simultaneously improve the ethical norms and biosafety assessment systems of gene editing and create a whole chain breeding mode of ‘gene design–precision selection–ecological adaptation–industry empowerment’ based on industrial applications, eventually creating a modern beef cattle breeding system based on gene technology, germplasm innovation, precision selection, and resistance enhancement, thus promoting the leapfrog development of the beef cattle breeding industry (Figure 4).

## 5. Breeding New Lines of Dual-Purpose Cattle

**Figure 5:**
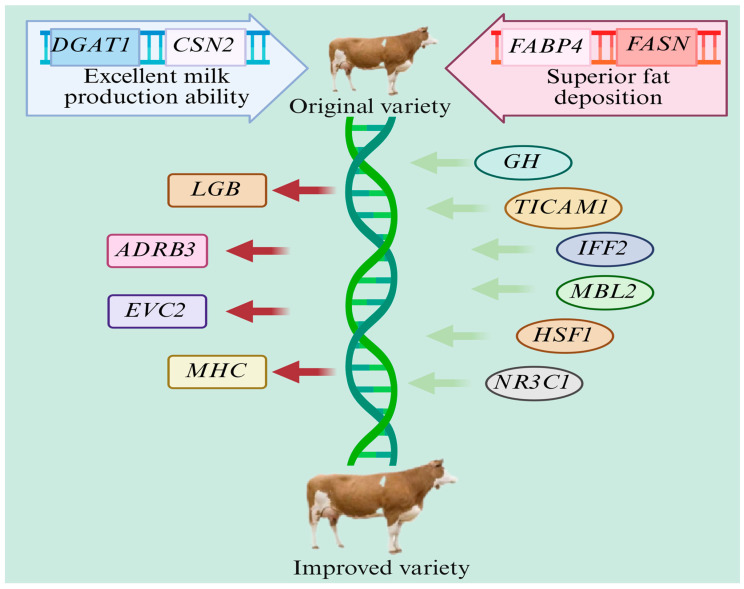
Schematic diagram of the breeding of a new dual-purpose cattle line (green arrows represent insertion, and red arrows represent deletion).

All genes related to lactation and meat production mentioned earlier have similar applications in dual-purpose cattle. Therefore, we will next discuss feasible ideas for the future breeding of dual-purpose cattle. The breeding of new lines of dual-purpose cattle requires a combination of genomics and gene editing to ensure a balance of traits based on the existing breeds. Scientists should first screen cattle with a high milk protein content (e.g., a β-casein B allele phenotype) and high-quality intramuscular fat deposition (e.g., a high expression of the *FABP4* gene) as core parents. The rapid growth characteristics (*GH* gene variation) of Charolais cattle or the antioxidant components in milk fat (regulated by the *SCD* gene) of Guernsey cattle should be introduced through hybridization. At the same time, tick-resistant genes (such as the TICAM1 pathway) of African Sanga cattle or cold-resistant genes (such as the allelic variation in *UCP2* [112]) of Nordic cattle breeds should be integrated into the genes of dual-purpose cattle to enhance their environmental adaptability. Single-cell transcriptome technology should be utilized to decipher the key regulatory networks of mammary epithelial cells and skeletal muscle stem cells. The targeted editing of milk production-related genes (for example, knocking out the *LGB* gene to reduce the allergenicity of β-lactoglobulin and inserting the *CSN3* promoter to enhance the expression of κ-casein) and meat production-related genes (such as activating the imprinted regulatory region of *IGF2* to improve muscle differentiation efficiency and inhibiting the *ADRB3* gene to reduce excessive fat deposition) should be carried out. Simultaneously, disease resistance modules (such as using the *MBL2* gene to enhance innate immunity and the *SLC11A1* mutant to improve anti-tuberculosis ability) and stress response pathways (such as the heat-resistant allele of *HSF1* [113] and its optimization [114]) should be integrated. The off-target risk of CRISPR/Cas12a and base editing technologies should be reduced. Embryo chimerism technology should be used to rapidly obtain gene-chimeric populations. Nanopore real-time sequencing should be utilized to monitor epigenetic modifications (such as the influence of DNA methylation on milk production traits [115]) and to simulate extreme climates in a controlled-environment chamber to verify the adaptive phenotypes. Finally, gut microbiota transplantation should be combined with dynamic metabolomic analysis to screen new cattle lines with synergistically improved milk and meat performance, stress resistance, and genetic stability [116] (Figure 5).

## 6. Discussion and Conclusions

With the continuous progress of science and technology and the increasing diversification of market demands, the breeding of new lines of cattle has shown a series of new development trends [117]. The in-depth integration of molecular and traditional breeding has become an important future direction for the livestock industry. Traditional breeding methods first involve the selection of appearance or quality characteristics and then the use of techniques such as crossbreeding improvement and variety selection to improve cattle breeds [118]. However, traditional breeding methods have limitations, such as being time-consuming and having poor predictability and low accuracy. With the rapid development of modern biotechnology and genomics, molecular breeding techniques have emerged. Molecular breeding enables the precise selection and utilization of superior genes by locating and determining genes for economically important traits in cattle. The use of genome-wide selection techniques can improve the efficiency and accuracy of gene selection by analyzing genome-wide genetic markers to accurately assess the genetic value of individuals during the early growth stages of cattle [10]. Combining molecular breeding techniques with traditional breeding methods can greatly accelerate breeding progress and improve breeding results.

Mining the good genes of local breeds is also an important trend in the breeding of new lines of cattle. Many countries and regions have rich resources of local cattle breeds, and these local breeds are undergoing long-term natural selection as well as artificial selection and are forming many excellent traits, such as high disease resistances, high roughage-degradation efficiencies, and excellent meat qualities. *China’s Xiangxi yellow cattle* are adapted to the grazing environment of the grassy hills and slopes in the Western Hunan region of China and are characterized by their tolerance towards rough fodder and excellent meat quality [119]. Through molecular biology technology, promising genes of local breeds can be explored and studied, and these genes can be introduced into the breeding of new lines of cattle to enrich their genetic diversity and improve their adaptability and production performance; at the same time, this can also help to protect and utilize the resources of local cattle breeds.

As the public awareness of food safety and health continues to rise, there is an urgent need to develop cattle breeds with enhanced disease resistance and superior meat and milk quality. In terms of disease resistance, technologies such as gene editing and molecular marker-assisted selection enable the identification and breeding of cattle with genetic resistance to common diseases, including foot-and-mouth disease, bovine viral diarrhea, and brucellosis. This approach reduces the risk of disease outbreaks, minimizes drug use, and safeguards both herd health and food safety [120]. Regarding meat and milk quality, greater emphasis is being placed on breeding cattle with desirable traits such as vibrant meat color, rich marbling, balanced fat content, and high milk fat and protein contents to meet the consumer demand for premium products. Furthermore, intelligent breeding techniques are expected to play an increasingly vital role in the development of new cattle breeds. With the rapid advancement of the Internet of Things (IoT), big data, and artificial intelligence (AI), intelligent breeding technologies now enable the real-time monitoring and precise regulation of cattle growth environments, feeding management, and health status, offering significant support for breeding efforts [121]. For instance, sensors can be used to monitor barn conditions such as temperature, humidity, and air quality in real time, while automated systems can adjust ventilation, heating, and cooling equipment to create an optimal environment for cattle growth. Additionally, big data analytics can process growth, reproduction, and health data to provide a scientific basis for farming decisions, enhancing both operational efficiency and management standards [122]. Furthermore, AI technologies, including image and voice recognition, can facilitate disease diagnosis and early warning systems by promptly detecting abnormalities in the herd, enabling proactive preventive and curative measures [123].

Despite the numerous issues associated with gene editing, such as the challenges faced by animals in their survival after mutagenesis, the insufficient accuracy of gene editing, consumers’ resistance towards animal products resulting from gene editing, and the complex ethical and regulatory environment, we still firmly believe that gene editing will offer substantial assistance in the creation of new cattle breeds [124,125]. The development of new cattle breeds is not only a significant leap in livestock farming technology but also an innovative response to global challenges. By integrating gene editing, whole-genome selection, and intelligent biological breeding technologies, not only can the efficiency and quality of meat and dairy production be significantly improved, but global carbon emission reduction targets can also be achieved by optimization of the feed conversion rate and reductions in methane emissions. Under the multiple pressures of population growth, climate change, and resource constraints, if the required cattle breeds can be efficiently created through gene editing, global food security will be enhanced, and the development of sustainable agriculture will be promoted. Furthermore, this will create a new avenue for the livestock industry to contribute to climate governance. In addition, the disease resistance and environmental adaptability of these new cattle breeds will also provide solutions for livestock production in ecologically vulnerable areas such as tropical and arid regions, helping to overcome resource constraints. Ultimately, this ‘genetic revolution’ in the field of cattle breeding represents a major effort made by humanity to ensure a sustainable future.

## Figures and Tables

**Table 1:** Classification of genes affecting traits in dairy cattle.

Gene Names	Main Function	References
*FABP3*, *PPARG*, *AGPAT6*, *SCD1*, *DGAT1*, *SREBP1*	Regulation of milk fat metabolism	[31,32,33,34,35]
*CSN2*, *CSN3*, *LGB*	Regulation of milk protein metabolism	[36,37]
*IQGAP2*	Regulation of lactose metabolism	[38]
*FSHR*, *PRLR*, *BMP15*, *KISS1*	Influence reproductive function	[28,39,40,41]
*CD14*, *CXCR1*, *MBL1*	Modulation of immune function	[42,43,44]
*HSP70*, *NR3C1*	Improvement in stress capacity	[45,46]
*TERT*	Regulation of rate of aging	[47]

**Table 2:** Functions of genes affecting traits in beef cattle.

Gene Name	Trait	Reference
*ADIPOQ*	Marble pattern	[86]
*PRKAG3*	Muscle pH	[87]
	Cartilage length	[88]
*LEP*	Fatty acid composition	[89]
	Eye muscle area	[90]
	Muscle pH	[91]
	Marble pattern	[92]
*CAST*	Muscle iron content	[93]
	Muscle pH	[94]
	Juiciness	[95]
*FASN*	MUFA	[96]
	SFA	[97]
	C14:0	[98]
	C16:0	[99]
	C18:0	[100]
	Carcass weight	[101]
*MC4R*	Muscle pH	[87]
	Carcass weight	[102]
	Marble pattern	[103]

## Data Availability

No new data were created or analyzed in this study.

## References

[1] Thornton P.K. (2010). Livestock production: Recent trends, future prospects. Philos. Trans. R. Soc. London. Ser. B Biol. Sci..

[2] Oleinik S.A., Maltsev A.E., Filatov D.A. (2024). Comparative assessment of the productive qualities of Holstein and Jersey dairy cattle. BIO Web. Conf..

[3] Pascottini O.B., Crowe A.D., Ramil U.Y., Hostens M., Opsomer G., Crowe M.A. (2025). Perspectives in cattle reproduction for the next 20 years—A European context. Theriogenology.

[4] Scott B.A., Mariam M.H., Tiezzi F., Berg I.v.d., Maltecca C., Pryce J.E. (2024). Optimizing genetic diversity in Australian Holsteins and Jerseys: A comparative analysis of whole-genome and regional inbreeding depression effects. J. Dairy Sci..

[5] Berry D.P., Wall E., Pryce J.E. (2014). Genetics and genomics of reproductive performance in dairy and beef cattle. Animal.

[6] Ahmad S., Agung I., Hendra H., Jasmadi, Ainsyar H.M., Angger S.A., Erma S.A., Hilda N., Taufik K., Guna D.I.N. (2022). Effects of various macroalgae species on methane production, rumen fermentation, and ruminant production: A meta-analysis from in vitro and in vivo experiments. Anim. Feed Sci. Technol..

[7] Mikkola M., Desmet K.L.J., Kommisrud E., Riegler M.A. (2024). Recent advancements to increase success in assisted reproductive technologies in cattle. Anim. Reprod..

[8] Mueller M.L., Van Eenennaam A.L. (2022). Synergistic power of genomic selection, assisted reproductive technologies, and gene editing to drive genetic improvement of cattle. CABI Agric. Biosci..

[9] Xu L., Bickhart D.M., Cole J.B., Schroeder S.G., Song J., Tassell C.P.V., Sonstegard T.S., Liu G.E. (2015). Genomic signatures reveal new evidences for selection of important traits in domestic cattle. Mol. Biol. Evol..

[10] Alves F.J.G., Alves S.D., Macedo M.L.F., Pinto d.M.T., Simielli F.L.F., Santos S.D.B.d., Roberto C., Galvão A.L. (2022). Sustainable Intensification of Beef Production in the Tropics: The Role of Genetically Improving Sexual Precocity of Heifers. Animals.

[11] Alves F.J.G., Elisa P., Iana S.P., Soares C.G., Macedo M.L.F., Zerlotti M.M.E., Fernando B., Roberto C., Galvão d.A.L. (2022). Current applications and perspectives of genomic selection in Bos indicus (Nellore) cattle. Livest. Sci..

[12] Zhang Z., Erbe M., He J., Ober U., Gao N., Zhang H., Simianer H., Li J. (2015). Accuracy of whole-genome prediction using a genetic architecture-enhanced variance-covariance matrix. G3.

[13] Brøndum R.F., Su G., Janss L., Sahana G., Guldbrandtsen B., Boichard D., Lund M.S. (2015). Quantitative trait loci markers derived from whole genome sequence data increases the reliability of genomic prediction. J. Dairy Sci..

[14] John v.d.O., Constantinos P. (2023). The genome editing revolution. Trends Biotechnol..

[15] Christian M., Cermak T., Doyle E.L., Schmidt C., Zhang F., Hummel A., Bogdanove A.J., Voytas D.F. (2010). Targeting DNA double-strand breaks with TAL effector nucleases. Genetics.

[16] Cong L., Ran F.A., Cox D., Lin S., Barretto R., Habib N., Hsu P.D., Wu X., Jiang W., Marraffini L.A. (2013). Multiplex Genome Engineering Using CRISPR/Cas Systems. Science.

[17] Magdalena H., Daniel L., Joanna Z., Ryszard S. (2017). CRISPR/Cas9 Immune System as a Tool for Genome Engineering. Arch. Immunol. Ther. Exp..

[18] Kuznetsov V., Revina G., Astashenkova L. (2020). Additive-Polygenic Inheritance of Reproductive System Diseases in Holstein Cows in Subpopulations. International Scientific Conference the Fifth Technological Order: Prospects for the Development and Modernization of the Russian Agro-Industrial Sector (TFTS 2019).

[19] Dong F., Xie K., Chen Y., Yang Y., Mao Y. (2017). Polycistronic tRNA and CRISPR guide-RNA enables highly efficient multiplexed genome engineering in human cells. Biochem. Biophys. Res. Commun..

[20] Feng X., Zhao D., Zhang X., Ding X., Bi C. (2018). CRISPR/Cas9 assisted Multiplex Genome Editing Technique in *Escherichia coli*. Biotechnol. J..

[21] Tetsushi S., Ayami N., Satoshi K., Kazuaki C., Takashi Y. (2014). Multiplex genome engineering in human cells using all-in-one CRISPR/Cas9 vector system. Sci. Rep..

[22] Kor S.D., Chowdhury N., Keot A.K., Yogendra K., Chikkaputtaiah C., Sudhakar Reddy P. (2022). RNA Pol III promoters-key players in precisely targeted plant genome editing. Front. Genet..

[23] Nie L., Thakur M.D., Wang Y., Su Q., Zhao Y., Feng Y. (2010). Regulation of U6 Promoter Activity by Transcriptional Interference in Viral Vector-Based RNAi. Genom. Proteom. Bioinform..

[24] Muntazir M., Aejaz A.D., Milan S., Anshika T., Nancy B., Umer B., Ahmad B.B., Abbu Z., Sajad A., TanvirUlHassan D. (2021). CRISPR-Based Genome Editing Tools: Insights into Technological Breakthroughs and Future Challenges. Genes.

[25] Kishimoto T., Nishimura K., Morishita K., Fukuda A., Miyamae Y., Kumagai Y., Sumaru K., Nakanishi M., Hisatake K., Sano M. (2024). An engineered ligand-responsive Csy4 endoribonuclease controls transgene expression from Sendai virus vectors. J. Biol. Eng..

[26] Haurwitz R.E., Sternberg S.H., Doudna J.A. (2012). Csy4 relies on an unusual catalytic dyad to position and cleave CRISPR RNA. EMBO J..

[27] Zalatan J.G., Lee M.E., Almeida R., Gilbert L.A., Whitehead E.H., Russa M.L., Tsai J.C., Weissman J.S., Dueber J.E., Qi L.S. (2015). Engineering Complex Synthetic Transcriptional Programs with CRISPR RNA Scaffolds. Cell.

[28] Cuellar C.J., Amaral T.F., Rodriguez-Villamil P., Ongaratto F., Martinez D.O., Labrecque R., Losano J.D.D.A., Estrada-Cortés E., Bostrom J.R., Martins K. (2024). Consequences of gene editing of PRLR on thermotolerance, growth, and male reproduction in cattle. FASEB BioAdvances.

[29] Zhao Y., Yang L., Su G., Wei Z., Liu X., Song L., Hai C., Wu D., Hao Z., Wu Y. (2022). Growth Traits and Sperm Proteomics Analyses of Myostatin Gene-Edited Chinese Yellow Cattle. Life.

[30] Wang Y., Bi D., Qin G., Song R., Yao J., Cao C., Zheng Q., Hou N., Wang Y., Zhao J. (2020). Cytosine Base Editor (hA3A-BE3-NG)-Mediated Multiple Gene Editing for Pyramid Breeding in Pigs. Front. Genet..

[31] Kumar D.P., Shubham G., Kumar M.S., Reena A., Manishi M., Kumar N.S., Periasamy K., Singh K.R. (2016). Identification of polymorphism in fatty acid binding protein 3 (FABP3) gene and its association with milk fat traits in riverine buffalo (*Bubalus bubalis*). Trop. Anim. Health Prod..

[32] Wang C., Zhao J., Feng X., Zhao W., Ma R., Yu B., Xue L., Wang H., Chen Y., Zhang J. (2024). bta-miR-224 regulates milk fat metabolism by targeting FABP4 in bovine mammary epithelial cells. Genomics.

[33] Zhou F., Xue J., Shan X., Qiu L., Miao Y. (2022). Functional roles for AGPAT6 in milk fat synthesis of buffalo mammary epithelial cells. Anim. Biotechnol..

[34] Ramos M.C.G., Souza F.P.A.d., Diniz P.M.G.C., Cruz R.I., Ferraz L.F.C., Thalia Z., Vercesi F.A.E., Tomita B.F.Â., Santos C.M.R., Sundfeld G.M.A. (2023). Phenotypic variation in milk fatty acid composition and its association with stearoyl-CoA desaturase 1 (SCD1) gene polymorphisms in Gir cows. J. Anim. Breed. Genet. = Z. Fur Tierz. Und Zucht..

[35] Li N., Zhao F., Wei C., Liang M., Zhang N., Wang C., Li Q.-Z., Gao X.-J. (2014). Function of SREBP1 in the Milk Fat Synthesis of Dairy Cow Mammary Epithelial Cells. Int. J. Mol. Sci..

[36] Amalfitano N., Mota L.F.M., Rosa G.M., Cecchinato A., Bittante G. (2022). Role of CSN2, CSN3, and BLG genes and the polygenic background in the cattle milk protein profile. J. Dairy Sci..

[37] Molee A., Poompramun C., Mernkrathoke P. (2015). Effect of casein genes—Beta-LGB, DGAT1, GH, and LHR—On milk production and milk composition traits in crossbred Holsteins. Genet. Mol. Res. GMR.

[38] Zhang J., Yang G., Zha X., Ma X., La Y., Wu X., Guo X., Chu M., Bao P., Yan P. (2024). Polymorphisms Within the IQGAP2 and CRTAC1 Genes of Gannan Yaks and Their Association with Milk Quality Characteristics. Foods.

[39] Pambuko G., Vanessa R., Widyastuti R., Prastowo S. (2024). FSHR gene polymorphism and its association to reproductive traits in Friesian Holstein cattle. IOP Conf. Ser. Earth Environ. Sci..

[40] Yang X., Wang Z., Chen Y., Ding H., Fang Y., Ma X., Liu H., Guo J., Zhao J., Wang J. (2024). ALKBH5 Reduces BMP15 mRNA Stability and Regulates Bovine Puberty Initiation Through an m6A-Dependent Pathway. Int. J. Mol. Sci..

[41] Umesh S., Rani A., Sushil K., Rajib D., Thiruvothur V.R., Shaily S., Singh S.G., Bharat S.R., Kotikalapudi S.M., Nitin V.R.P. (2020). Association of bovine KISS1 single nucleotide polymorphisms with reproductive traits in Indian Cattle. Reprod. Domest. Anim. = Zuchthyg..

[42] Gupta J.P., Bhushan B., Asaf V.N.M., Kumar A., Ranjan S., Panigrahi M., Kumar A., Kumar P. (2018). Association and expression analysis of single nucleotide polymorphisms of CD14 gene with somatic cell score in crossbred cattle. Gene Rep..

[43] Muslimova Z., Abdualiyeva A., Shaugimbayeva N., Orynkhanov K., Ussenbekov Y. (2024). Genotyping of Holstein Cows by SELL, MX1 and CXCR1 Gene Loci Associated With Mastitis Resistance. Reprod. Domest. Anim. = Zuchthyg..

[44] Kamaldeep, Magotra A., Pander B.L., Dalal D.S., Malik B.S., Garg A.R., Malik A. (2021). Evaluation of candidate genotype of immune gene MBL1 associated with udder health and performance traits in dairy cattle and buffalo of India. Trop. Anim. Health Prod..

[45] Lim J.W., Lee J.H., Nejad J.G., Lee H.G. (2024). Effects of L-leucine and sodium acetate on milk protein synthesis under heat stress conditions in bovine mammary epithelial cells in vitro. J. Therm. Biol..

[46] Vilela P.B., Bonvino S.N., Afonso d.F.L., Zerlotti M.M.E., Silveira R.E., Paro P.C.C. (2021). Expression of candidate genes for residual feed intake in tropically adapted Bos taurus and Bos indicus bulls under thermoneutral and heat stress environmental conditions. J. Therm. Biol..

[47] Xu L., Idrees M., Joo M.D., Sidrat T., Wei Y., Song S.H., Li K.L., Kong I.K. (2021). Constitutive Expression of TERT Enhances β-Klotho Expression and Improves Age-Related Deterioration in Early Bovine Embryos. Int. J. Mol. Sci..

[48] Oleszycka E., Kwiecień K., Grygier B., Cichy J., Kwiecińska P. (2024). The many faces of DGAT1. Life Sci..

[49] (2009). Thematic Review Series: Glycerolipids. DGAT enzymes and triacylglycerol biosynthesis. Int. News Fats Oils Relat. Mater. Inform.

[50] Elzaki S., Korkuć P., Arends D., Reissmann M., Brockmann G.A. (2022). Effects of DGAT1 on milk performance in Sudanese Butana × Holstein crossbred cattle. Trop. Anim. Health Prod..

[51] Zahoor K.M., Yulin M., Jiaying M., Jianxin X., Yue L., Shuai L., Adnan K., Muhammad K.I., Zhijun C. (2021). Association of DGAT1 With Cattle, Buffalo, Goat, and Sheep Milk and Meat Production Traits. Front. Vet. Sci..

[52] Mou M.A., Deb G.K., Hridoy M.F.A., Alam M.A., Barai H.R., Haque M.A., Bhuiyan M.S.A. (2024). Detection of Polymorphisms in FASN, DGAT1, and PPARGC1A Genes and Their Association with Milk Yield and Composition Traits in River Buffalo of Bangladesh. Animals.

[53] Li J., Liu S., Li Z., Zhang S., Hua G., Salzano A., Campanile G., Gasparrini B., Liang A., Yang L. (2018). DGAT1 polymorphism in Riverine buffalo, Swamp buffalo and crossbred buffalo. J. Dairy Res..

[54] Spelman R.J., Ford C.A., McElhinney P., Gregory G.C., Snell R.G. (2002). Characterization of the DGAT1 Gene in the New Zealand Dairy Population. J. Dairy Sci..

[55] Bovenhuis H., Visker M.H.P.W., Poulsen N.A., Sehested J., Valenberg H.J.F.v., Arendonk J.A.M.v., Larsen L.B., Buitenhuis A.J. (2016). Effects of the diacylglycerol o-acyltransferase 1 (DGAT1) K232A polymorphism on fatty acid, protein, and mineral composition of dairy cattle milk. J. Dairy Sci..

[56] Bobbo T., Tiezzi F., Penasa M., Marchi M.D., Cassandro M. (2018). Short communication: Association analysis of diacylglycerol acyltransferase (DGAT1) mutation on chromosome 14 for milk yield and composition traits, somatic cell score, and coagulation properties in Holstein bulls. J. Dairy Sci..

[57] Argov-Argaman N., Mida K., Cohen B.-C., Visker M., Hettinga K. (2017). Milk fat content and DGAT1 genotype determine lipid composition of the milk fat globule membrane. PLoS ONE.

[58] (2011). DGAT1-dependent triacylglycerol storage by macrophages protects mice from diet-induced insulin resistance and inflammation. J. Clin. Investig..

[59] El-Komy S.M., Saleh A.A., Abdel-Hamid T.M., El-Magd M.A. (2020). Association of GHR Polymorphisms with Milk Production in Buffaloes. Animals.

[60] Sakamoto K., Komatsu T., Kobayashi T., Rose M.T., Aso H., Hagino A., Obara Y. (2005). Growth hormone acts on the synthesis and secretion of α-casein in bovine mammary epithelial cells. J. Dairy Res..

[61] Maj A., Oprzadek J., Oprzadek A., Dymnicki E., Zwierzchowski L. (2004). Polymorphism in the 5′-noncoding region of the bovine growth hormone receptor gene and its association with meat production traits in cattle. Anim. Res..

[62] Andrzej M., Lech Z. (2006). Molecular evolution of coding and non-coding sequences of the growth hormone receptor (GHR) gene in the family Bovidae. Folia Biol..

[63] Waters S.M., McCabe M.S., Howard D.J., Giblin L., Magee D.A., MacHugh D.E., Berry D.P. (2011). Associations between newly discovered polymorphisms in the Bos taurus growth hormone receptor gene and performance traits in Holstein-Friesian dairy cattle. Anim. Genet..

[64] Sanchez M.P., Govignon-Gion A., Ferrand M., Gelé M., Pourchet D., Amigues Y., Fritz S., Boussaha M., Capitan A., Rocha D. (2016). Whole-genome scan to detect quantitative trait loci associated with milk protein composition in 3 French dairy cattle breeds. J. Dairy Sci..

[65] Sarah B., Jong-Joo K., Sirja M., Anne S.-K., Anne C., Paulette B., Nadine C., Christine F., Bernard G., Dave J. (2003). Molecular dissection of a quantitative trait locus: A phenylalanine-to-tyrosine substitution in the transmembrane domain of the bovine growth hormone receptor is associated with a major effect on milk yield and composition. Genetics.

[66] Rahmatalla S.A., Müller U., Strucken E.M., Reissmann M., Brockmann G.A. (2011). The F279Y polymorphism of the GHR gene and its relation to milk production and somatic cell score in German Holstein dairy cattle. J. Appl. Genet..

[67] Ma Y.N., He P.J., Zhu J., Lei Z.M., Liu Z., Wu J.P. (2013). The effect of polymorphism F279Y of GHR gene on milk production trait in Chinese Holstein cattle. Zhongguo Ying Yong Sheng Li Xue Za Zhi = Zhongguo Yingyong Shenglixue Zazhi = Chin. J. Appl. Physiol..

[68] Perry J.K., Mohankumar K.M., Emerald B.S., Mertani H.C., Lobie P.E. (2008). The contribution of growth hormone to mammary neoplasia. J. Mammary Gland Biol. Neoplasia.

[69] Cui Y., Sun X., Jin L., Yu G., Li Q., Gao X., Ao J., Wang C. (2017). MiR-139 suppresses β-casein synthesis and proliferation in bovine mammary epithelial cells by targeting the GHR and IGF1R signaling pathways. BMC Vet. Res..

[70] Cordero A., Pellegrini P., Sanz-Moreno A., Trinidad E.M., Serra-Musach J., Deshpande C., Dougall W.C., Pujana M.A., González-Suárez E. (2016). Rankl Impairs Lactogenic Differentiation Through Inhibition of the Prolactin/Stat5 Pathway at Midgestation. Stem Cells.

[71] Oakes S.R., Rogers R.L., Naylor M.J., Ormandy C.J. (2008). Prolactin regulation of mammary gland development. J. Mammary Gland Biol. Neoplasia.

[72] Hennighausen L., Robinson G.W., Wagner K.-U., Liu X. (1997). Prolactin Signaling in Mammary Gland Development. J. Biol. Chem..

[73] Mapes J., Li Q., Kannan A., Anandan L., Laws M., Lydon J.P., Bagchi I.C., Bagchi M.K. (2017). CUZD1 is a critical mediator of the JAK/STAT5 signaling pathway that controls mammary gland development during pregnancy. PLoS Genet..

[74] Mehmannavaz Y., Amirinia C., Bonyadi M., Torshizi R. (2009). Effects of bovine prolactin gene polymorphism within exon 4 on milk related traits and genetic trends in Iranian Holstein bulls. Afr. J. Biotechnol..

[75] Elena I.D., Eugeniu M.A., Valentin M.C., Ionel N.R., Toma C.L., Mihai C., Cristian G.A. (2023). Polymorphism of the Prolactin (PRL) Gene and Its Effect on Milk Production Traits in Romanian Cattle Breeds. Vet. Sci..

[76] Corina P., Viorel H., Ionica I., Luminita C. (2022). Etiology of Mastitis and Antimicrobial Resistance in Dairy Cattle Farms in the Western Part of Romania. Antibiotics.

[77] Salgado-Lora M.G., Medina-Estrada I., López-Meza J.E., Ochoa-Zarzosa A. (2020). Prolactin and Estradiol are Epigenetic Modulators in Bovine Mammary Epithelial Cells during Staphylococcus aureus Infection. Pathogens.

[78] Antonio B.M.M., Guadalupe S.L.M., Edmundo L.M.J., Alejandra O.Z. (2022). Prolactin regulates H3K9ac and H3K9me2 epigenetic marks and miRNAs expression in bovine mammary epithelial cells challenged with *Staphylococcus aureus*. Front. Microbiol..

[79] Beishova I., Belaya A., Kuzhebayeva U., Ulyanova T., Ulyanov V., Beishov R., Ginayatov N., Kovalchuk A., Kharzhau A., Sidarova A. (2024). Association of polymorphic variants of prolactin (PRL) and beta-lactoglobulin (BLG) genes with resistance/susceptibility to mastitis in holstein cows. Braz. J. Biol. = Rev. Brasleira De Biol..

[80] Jiang L., Liu J., Sun D., Ma P., Ding X., Yu Y., Zhang Q. (2010). Genome wide association studies for milk production traits in Chinese Holstein population. PLoS ONE.

[81] Liang Z., Prakapenka D., VanRaden P.M., Jiang J., Ma L., Da Y. (2023). A Million-Cow Genome-Wide Association Study of Three Fertility Traits in U.S. Holstein Cows. Int. J. Mol. Sci..

[82] Mohamed A.I., Xubin L., Mudasir N., Idriss A.A.A., Tianle X., Husien Y.M., Yongjiang M., Zhangping Y. (2021). Genome-Wide Association Study Identifies Candidate Genes Associated with Feet and Leg Conformation Traits in Chinese Holstein Cattle. Animals.

[83] Klein S.L., Scheper C., May K., König S. (2020). Genetic and nongenetic profiling of milk β-hydroxybutyrate and acetone and their associations with ketosis in Holstein cows. J. Dairy Sci..

[84] Freebern E., Santos D.J., Fang L., Jiang J., Parker Gaddis K.L., Liu G.E., VanRaden P.M., Maltecca C., Cole J.B., Ma L. (2020). GWAS and fine-mapping of livability and six disease traits in Holstein cattle. BMC Genom..

[85] Nayeri S., Schenkel F., Fleming A., Kroezen V., Sargolzaei M., Baes C., Cánovas A., Squires J., Miglior F. (2019). Genome-wide association analysis for β-hydroxybutyrate concentration in Milk in Holstein dairy cattle. BMC Genet..

[86] Choi Y., Davis M.E., Chung H. (2015). Effects of genetic variants in the promoter region of the bovine adiponectin (ADIPOQ) gene on marbling of Hanwoo beef cattle. Meat Sci..

[87] Ribeca C., Bonfatti V., Cecchinato A., Albera A., Gallo L., Carnier P. (2014). Effect of polymorphisms in candidate genes on carcass and meat quality traits in double muscled Piemontese cattle. Meat Sci..

[88] Gill J.L., Bishop S.C., McCorquodale C., Williams J.L., Wiener P. (2010). Associations between single nucleotide polymorphisms in multiple candidate genes and carcass and meat quality traits in a commercial Angus-cross population. Meat Sci..

[89] Orrù L., Cifuni G.F., Piasentier E., Corazzin M., Bovolenta S., Moioli B. (2010). Association analyses of single nucleotide polymorphisms in the LEP and SCD1 genes on the fatty acid profile of muscle fat in Simmental bulls. Meat Sci..

[90] Da Silva R.C.G., Ferraz J.B.S., Meirelles F.V., Eler J.P., Balieiro J.C.D.C., Cucco D.D.C., Mattos E.C., Rezende F.M.D., Silva S.D.L. (2012). Association of single nucleotide polymorphisms in the bovine leptin and leptin receptor genes with growth and ultrasound carcass traits in Nellore cattle. Genet. Mol. Res. GMR.

[91] Tian J., Zhao Z., Zhang L., Zhang Q., Yu Z., Li J., Yang R. (2013). Association of the leptin gene E2-169T>C and E3-299T>A mutations with carcass and meat quality traits of the Chinese Simmental-cross steers. Gene.

[92] Melucci L.M., Panarace M., Feula P., Villarreal E.L., Grigioni G., Carduza F., Soria L.A., Mezzadra C.A., Arceo M.E., Mazzucco J.P. (2012). Genetic and management factors affecting beef quality in grazing Hereford steers. Meat Sci..

[93] Casas E., Duan Q., Schneider M.J., Shackelford S.D., Wheeler T.L., Cundiff L.V., Reecy J.M. (2014). Polymorphisms in calpastatin and mu-calpain genes are associated with beef iron content. Anim. Genet..

[94] Cinzia R., Valentina B., Alessio C., Andrea A., Fabio M., Luigi G., Paolo C. (2013). Association of polymorphisms in calpain 1, (mu/I) large subunit, calpastatin, and cathepsin D genes with meat quality traits in double-muscled Piemontese cattle. Anim. Genet..

[95] SeungHwan L., SeungChang K., HanHa C., SooHyun C., HyeongCheol K., DaJeong L., BongHwan C., ChangGwan D., Aditi S., Gondro C. (2014). Mutations in calpastatin and micro -Calpain are associated with meat tenderness, flavor and juiciness in Hanwoo (Korean cattle): Molecular modeling of the effects of substitutions in the calpastatin/ micro -Calpain complex. Meat Sci..

[96] Joo K.H., Aditi S., Hyun L.S., Ho L.D., Jeong L.D., Min C.Y., Suk Y.B., Hwan L.S. (2017). Genetic association of PLAG1, SCD, CYP7B1 and FASN SNPs and their effects on carcass weight, intramuscular fat and fatty acid composition in Hanwoo steers (Korean cattle). Anim. Genet..

[97] Bartoň L., Bureš D., Kott T., Řehák D. (2016). Associations of polymorphisms in bovine DGAT1, FABP4, FASN, and PPARGC1A genes with intramuscular fat content and the fatty acid composition of muscle and subcutaneous fat in Fleckvieh bulls. Meat Sci..

[98] Bhuiyan M.S.A., Kim Y.K., Kim H.J., Lee D.H., Lee S.H., Yoon H.B., Lee S.H. (2018). Genome-wide association study and prediction of genomic breeding values for fatty-acid composition in Korean Hanwoo cattle using a high-density single-nucleotide polymorphism array 1. J. Anim. Sci..

[99] Mazzucco J.P., Goszczynski D.E., Ripoli M.V., Melucci L.M., Pardo A.M., Colatto E., Rogberg-Muñoz A., Mezzadra C.A., Depetris G.J., Giovambattista G. (2016). Growth, carcass and meat quality traits in beef from Angus, Hereford and cross-breed grazing steers, and their association with SNPs in genes related to fat deposition metabolism. Meat Sci..

[100] Dongyep O., Yoonseok L., Boomi L., Jungsou Y., Euiryong C., Younyoung K., Chaeyoung L. (2012). Fatty acid composition of beef is associated with exonic nucleotide variants of the gene encoding FASN. Mol. Biol. Rep..

[101] Rempel L.A., Casas E., Shackelford S.D., Wheeler T.L. (2012). Relationship of polymorphisms within metabolic genes and carcass traits in crossbred beef cattle. J. Anim. Sci..

[102] Liu H., Tian W., Zan L., Wang H., Cui H. (2010). Mutations of MC4R gene and its association with economic traits in Qinchuan cattle. Mol. Biol. Rep..

[103] Jiyeon S., Sang S.D., Do P.K., Kyo L.H., Sik K.H. (2012). Identification and analysis of MC4R polymorphisms and their association with economic traits of Korean cattle (Hanwoo). Mol. Biol. Rep..

[104] Haruna I.L., Ekegbu U.J., Ullah F., Amirpour-Najafabadi H., Zhou H., Hickford J.G.H. (2020). Genetic variations and haplotypic diversity in the Myostatin gene of New Zealand cattle breeds. Gene.

[105] Martínez A., Aldai N., Celaya R., Osoro K. (2010). Effect of breed body size and the muscular hypertrophy gene in the production and carcass traits of concentrate-finished yearling bulls. J. Anim. Sci..

[106] Raes K., De Smet S., Demeyer D. (2001). Effect of double-muscling in Belgian Blue young bulls on the intramuscular fatty acid composition with emphasis on conjugated linoleic acid and polyunsaturated fatty acids. Anim. Sci..

[107] Fiems L.O. (2012). Double Muscling in Cattle: Genes, Husbandry, Carcasses and Meat. Animals.

[108] Robinson D.L., Cafe L.M., McIntyre B.L., Geesink G.H., Barendse W., Pethick D.W., Thompson J.M., Polkinghorne R., Greenwood P.L. (2012). Production and processing studies on calpain-system gene markers for beef tenderness: Consumer assessments of eating quality. J. Anim. Sci..

[109] Alves D.D., Goes R.H.d.T.e.B.d., Mancio A.B. (2006). Maciez da carne bovina. Ciência Anim. Bras..

[110] Shackelford S.D., Koohmaraie M., Cundiff L.V., Gregory K.E., Rohrer G.A., Savell J.W. (1994). Heritabilities and phenotypic and genetic correlations for bovine postrigor calpastatin activity, intramuscular fat content, Warner-Bratzler shear force, retail product yield, and growth rate1. J. Anim. Sci..

[111] Geary T.W., McFadin E.L., MacNeil M.D., Grings E.E., Short R.E., Funston R.N., Keisler D.H. (2003). Leptin as a predictor of carcass composition in beef cattle. J. Anim. Sci..

[112] Wang Y., Yang W., Gui L., Wang H., Zan L. (2016). Association and expression analyses of the Ucp2 and Ucp3 gene polymorphisms with body measurement and meat quality traits in Qinchuan cattle. J. Genet..

[113] Molinari P.C., Bromfield J.J. (2023). Inflammatory responses of bovine endometrial epithelial cells are increased under in vitro heat stress conditions. J. Therm. Biol..

[114] Poleti M.D., DeRijk R.H., Rosa A.F., Moncau C.T., Oliveira P.S., Coutinho L.L., Eler J.P., Balieiro J.C.C. (2015). Genetic variants in glucocorticoid and mineralocorticoid receptors are associated with concentrations of plasma cortisol, muscle glycogen content, and meat quality traits in male Nellore cattle. Domest. Anim. Endocrinol..

[115] Wang L., Sun H.Z., Guan L.L., Liu J.X. (2019). Short communication: Relationship of blood DNA methylation rate and milk performance in dairy cows. J. Dairy Sci..

[116] Geng Q., Lin W., Yang L., Hu X., Qiu X. (2025). Rumen-protected guanidinoacetic acid improves growth performance in beef cattle under chronic heat stress by reshaping gut microbiota and modulating serum metabolism. Front. Microbiol..

[117] Tian R., Mahmoodi M., Tian J., Koshkoiyeh S.E., Zhao M., Saminzadeh M., Li H., Wang X., Li Y., Esmailizadeh A. (2024). Leveraging Functional Genomics for Understanding Beef Quality Complexities and Breeding Beef Cattle for Improved Meat Quality. Genes.

[118] Cole J.B., VanRaden P.M. (2018). Symposium review: Possibilities in an age of genomics: The future of selection indices 1. J. Dairy Sci..

[119] Chen D., Wang X., Guo Q., Deng H., Luo J., Yi K., Sun A., Chen K., Shen Q. (2022). Muscle Fatty Acids, Meat Flavor Compounds and Sensory Characteristics of Xiangxi Yellow Cattle in Comparison to Aberdeen Angus. Animals.

[120] Kasimanickam R., Ferreira J.C.P., Kastelic J., Kasimanickam V. (2025). Application of Genomic Selection in Beef Cattle Disease Prevention. Animals.

[121] Kaur D., Virk A.K. (2025). Smart neck collar: IoT-based disease detection and health monitoring for dairy cows. Discov. Internet Things.

[122] Schnidrig G.A., Struchen R., Schärrer S., Heim D., Hadorn D., Regula G.S., Paternoster G. (2025). Improved cattle farm classification: Leveraging machine learning and linked national datasets. Front. Vet. Sci..

[123] Kittichai V., Kaewthamasorn M., Arnuphaprasert A., Jomtarak R., Naing K.M., Tongloy T., Chuwongin S., Boonsang S. (2025). A deep contrastive learning-based image retrieval system for automatic detection of infectious cattle diseases. J. Big Data.

[124] Dilger A.C. (2019). Gene editing in livestock: Advancements, opportunities and challenges. J. Anim. Sci..

[125] Villamil P.R., Beaton B.P., Krisher R.L. (2024). Gene editing in livestock: Innovations and applications. Anim. Reprod..

